# Effect of using a kinetic wedge during the hallux dorsiflexion resistance test in asymptomatic individuals

**DOI:** 10.1186/s12891-024-07520-z

**Published:** 2024-05-24

**Authors:** Álvaro Gómez-Carrión, Rubén Sánchez-Gómez, José Manuel Reguera-Medina, Carlos Martínez-Sebastián, Salvador Márquez-Reina, Manuel Coheña-Jiménez, Gabriel Moisan

**Affiliations:** 1 Nursing Department, Faculty of Nursing, Physiotherapy, and Podiatry, Universidad Complutense de Madrid, Madrid, 28040 Spain; 2 Podiatry Degree, Clinical Sanipie, Utrera Sevilla, 41704 Spain; 3 Podiatry Department, Faculty of Nursing, Physiotherapy, and Podiatry, Universidad de Sevilla, 41009 Seville, Spain; 4 Department of Human Kinetics, Université du Québec À Trois-Rivières, Trois Rivières, Québec Canada; 5 IdISSC, Institute for Health Research. Hospital Clínico San Carlos, 28040 Madrid, Spain

**Keywords:** Foot, Biomechanical phenomena, Foot orthoses, Force, Physical examination

## Abstract

**Background:**

The hallux dorsiflexion resistance test is a frequently employed clinical maneuver for assessing the initiation of the windlass mechanism This maneuver involves dorsiflexion of the phalanx of the hallux, thereby evaluating plantarflexion of the first metatarsal, elevation of the medial longitudinal arch, and supination of the rearfoot. The windlass mechanism plays a crucial role in gait, and orthopedic devices, such as a kinetic wedge, which aims to facilitate its activation by increasing the hallux dorsiflexion. Although it is believed that facilitating the windlass mechanism with the kinetic wedge should be directly correlated with a decrease in hallux dorsiflexion resistance, its effects have yet to be characterized. Thus, this study aimed to determine the influence of a kinetic wedge on hallux dorsiflexion resistance in asymptomatic individuals.

**Methods:**

The sample comprised thirty participants (14 women and 16 men). A digital force gauge measured the force required to perform the hallux dorsiflexion resistance test during two conditions: barefoot and with a kinetic wedge. The Wilcoxon signed-rank test was used to compare the hallux dorsiflexion resistance between conditions.

**Results:**

A statistically significant reduction in force (10.54 ± 3.16N vs. 19.62 ± 5.18N, *p* < 0.001) was observed when using the kinetic wedge compared to the barefoot condition during the hallux dorsiflexion resistance test.

**Conclusion:**

The use of a kinetic wedge reduces the required force for performing the passive hallux dorsiflexion resistance test in asymptomatic individuals. Future studies should determine to what extent the kinetic wedge can attenuate the required force to dorsiflex the hallux in individuals with musculoskeletal disorders such as plantar fasciopathy and functional hallux limitus.

## Introduction

The first (1st) metatarsophalangeal joint (MTPJ), formed by the hallux and the first metatarsal head, plays a pivotal role during gait. During the push-off phase, when the hallux dorsiflexes, the windlass mechanism initiates which consequently raises the medial longitudinal arch (MLA) [1]. The MLA plays a crucial role in dampening and redistributing ground reaction forces during walking [2]. The plantar fascia is a key component of the windlass mechanism and is interconnected with the MLA. This mechanism supports the proper functioning of the foot during gait [3–5]. When the hallux dorsiflexes, the mechanism triggers the plantarflexion of the 1st metatarsal head, the elevation of the MLA, inversion of the rearfoot, and the external rotation of the leg [6, 7]. When the dorsiflexion of the 1st MTPJ is restricted, the initiation of this mechanism during gait is prevented, and thus the biomechanics of the lower limbs during gait are modified [8, 9]. The 1st MTPJ exhibits a range of motion spanning from 30 to 50 degrees during normal gait [10]. Hallux limitus is characterized by a 1st MTPJ range of motion below the physiologic range, which is linked to an inefficient gait profile [10, 11]. Currently, research suggests that there is a limited relationship between foot posture and dynamic foot function with muskuloskeletal injuries[12–15]. It should be included more as a multifactorial factor in injury [12, 16]. However, kinetic variables linked to tissue loading might be more promising in the occurrence of musculoskeletal injuries.

Clinicians and researchers use the hallux dorsiflexion resistance test to assess the force required to induce dorsiflexion of the 1st MTPJ [17, 18]. This test also allows clinicians and researchers to estimate the required force to elicit the windlass mechanism under static conditions. The test is deemed normal when the required force to passively dorsiflex the 1st MTPJ is low [19]. However, previous studies found that the qualitative version of this test, the Hubscher maneuver or the Jack’s test [18], do not effectively predict the 1st MTPJ dorsiflexion during walking, thus questioning its clinical utility [17, 20]. However, most clinical tests aiming at predicting the lower limbs’ biomechanics from static measurements have failed [21, 22]. Recent studies suggest that clinical tests assessing the force acting on joints, rather than movement or range of motion, are reliable and predictive of dynamic gait biomechanics [23–25]. One of these tests is the hallux dorsiflexion resistance test (also named the 1st MTPJ dorsiflexion resistance test) [19, 26].

Orthopedic shoes inserts are used to treat many foot musculoskeletal disorders, mainly by modulating the forces acting on the foot [26]. To facilitate the windlass mechanism, it is common practice in clinical and research settings to use orthopedic shoes inserts, such as a kinetic wedge (KW) placed under the first metatarsal. The KW is specifically designed to reduce ground reaction forces under the head of the first metatarsal and aims to facilitate the windlass mechanism [17, 27, 28]. There is evidence suggesting that the use of a KW improves the position of the first metatarsal (28). Considering this, the required force to dorsiflex the 1st MTPJ should be decreased with the KW. However, the effects of the KW on the required force to dorsiflex the 1st MTPJ during the hallux dorsiflexion resistance test remains unknown.

Thus, the objective of this study was to investigate the effects of a KW during the hallux dorsiflexion resistance test in asymptomatic individuals. The hypothesis was that the utilization of the KW would decrease the required force to induce a dorsiflexion of the 1st MTPJ during the test and that the test would present excellent interrater and intertrial reliability.

## Material and methods

The Bioethics and Biosafety Research & Transfer Committee of the University of Extremadura approved this study (ID: 89_2023). This study was carried out in accordance with the guidelines for human ethics, as outlined in the Declaration of Helsinki. All participants signed an informed consent form (in accordance with Organic Law 15/99 of 13 December). This observational repeated measures design study was conducted in accordance with the Strengthening the Reporting of Observational Studies in Epidemiology (STROBE) guidelines [29].

## Participants

The required sample size was calculated by the Statistical Unit of the Complutense University of Madrid, based on previous data of our research group [26]. A sample size of at least 30 participants was determined to be adequate to obtain a power of 80%, β = 20%, α = 0.05, and 95% confidence interval (CI) for the hallux dorsiflexion resistance with the KW. Fifteen additional participants were added considering habitual loss and that this study is part of a larger project and that data from 45 participants were already available.

The inclusion criteria were as follows: 1) Male and female of 18 years or older [19]; Range of motion of the 1st MTPJ greater or equal to 30 degrees, measured with a manual goniometer [30]; and 3) Foot Posture Index score between 0 and + 5 (rectus foot type) [31, 32]; 4) No reported lower limb musculoskeletal injury in the 12 months period prior to the study onset [33] and 5) No history of surgery to the 1st MTPJ and imbalance due to neurological involvement [19].

## Instruments, measurement procedures, and variables

**Fig. 1:**
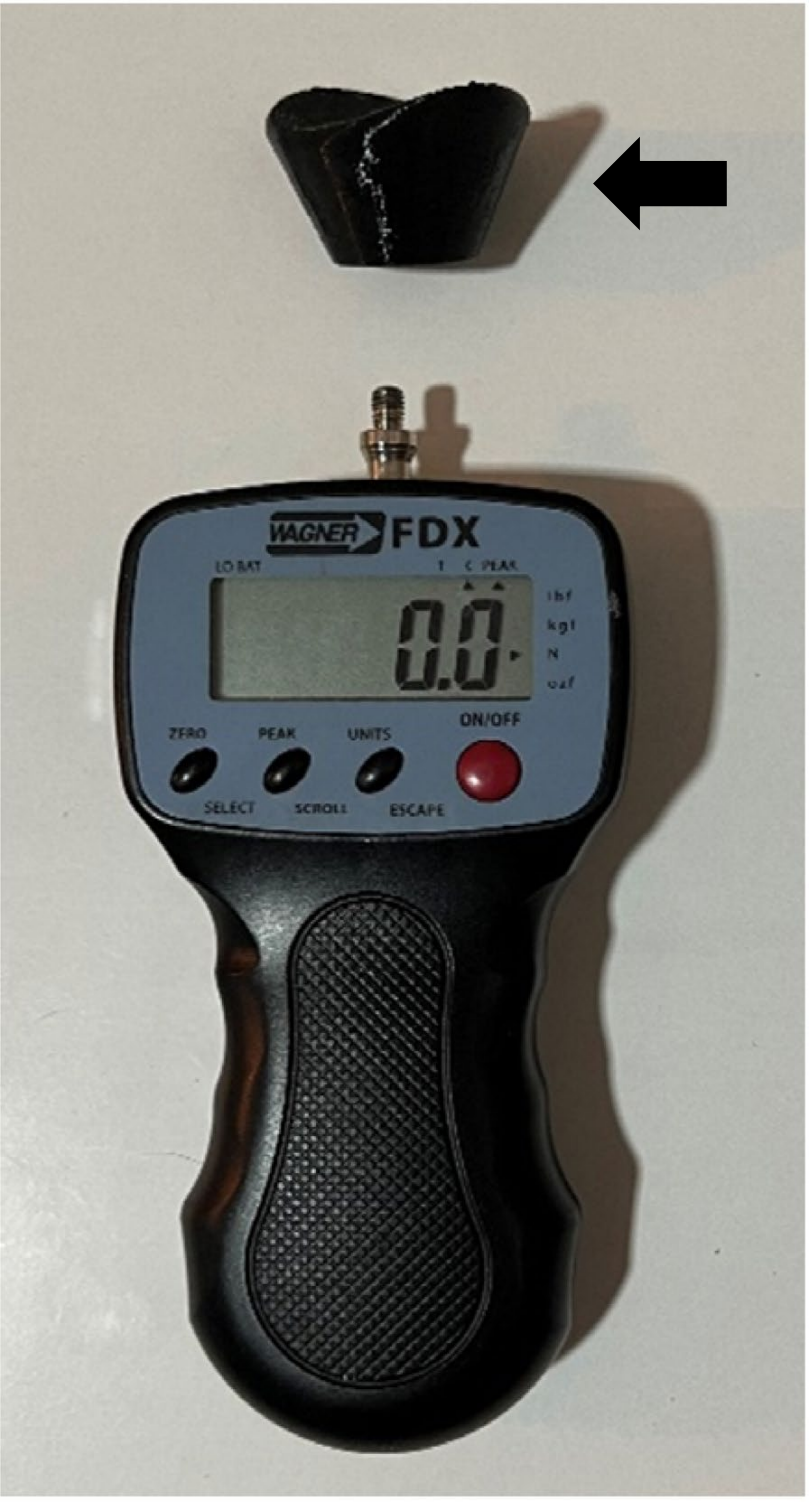
Digital force gauge FPX.®25 with a hallux adapter (Black arrow)

**Fig. 2:**
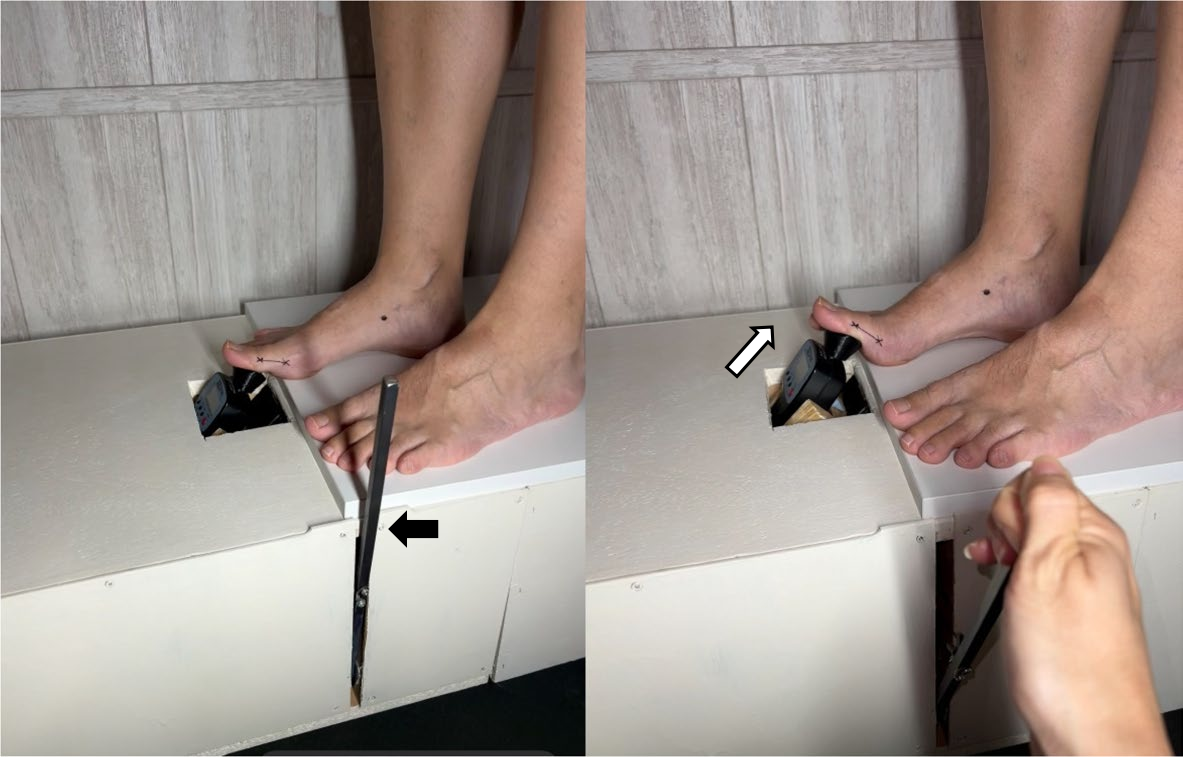
Sequence of use of the digital force gauge used to perform the hallux dorsiflexion resistance test. The Pulley system (Black arrow) allows the digital force gauge to move upwards over the hallux (White arrow)

The protocol used in this study was carried out in accordance with the research conducted by Gómez et al. [26].The Foot Posture Index was used to catalog the subjects' foot type. The Foot Posture Index (FPI) was assessed, a validated tool commonly employed for characterizing feet in numerous research studies. Based on the findings, feet can be categorized into three types: supinated, neutral, or pronated. The FPI demonstrates good reliability with an intraclass correlation coefficient ranging from 0.62 to 0.91. It consists of six criteria for assessment: palpation of the talus head, supra and infra malleolar curvature, prominence of the talar joint, arch height, position of the calcaneus in the frontal plane, and forefoot-rearfoot relationship. Subjects included in this study were required to have a score ranging from 0 to + 5[31, 32]. To perform the hallux dorsiflexion resistance test, participants were positioned on the digital force gauge with an adapter specifically designed for the hallux (FPX®25, Wagner Instruments®, Greenwich, CT, USA) [34–36] (see Fig. 1). The adapter was positioned on the proximal phalanx of the hallux to consistently use the same lever arm across participants. The digital force gauge was positioned on a piston with a rectilinear movement inclined at 45° relative to the proximal phalanx of the participants, ensuring a force perpendicular to the bone [37]. The device incorporated a lever and pulley system to manipulate the digital force gauge along the proximal phalanx, enabling the quantification of the required force, in Newtons, to perform the hallux dorsiflexion resistance test. The moment of activation of the windlass mechanism and the corresponding force, measured by the digital force gauge, were recorded simultaneously. The moment of activation of the windlass mechanism was determined with the use of a ruler. The mark on the navicular made it possible to establish the change in height and to register in the digital force gauge the force needed to raise the hallux. (6,26) (Fig. 2).

**Fig. 3:**
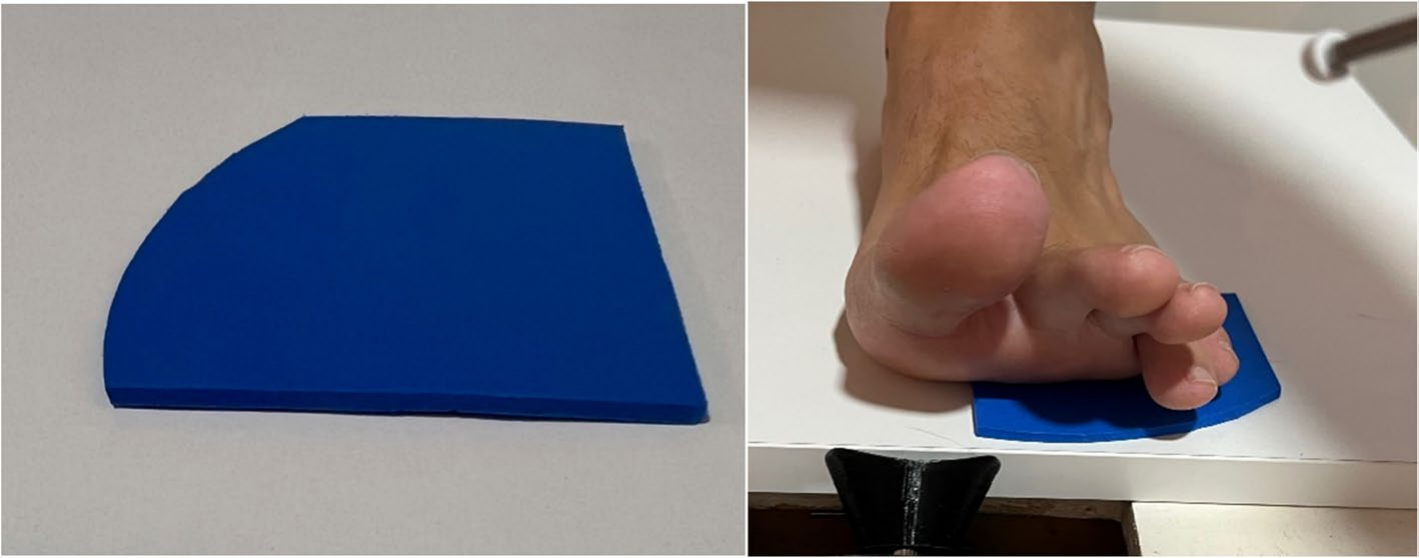
Kinetic wedge placed from second to fifth metatarsal leaving the first metatarsal head free

The measurements were conducted by two researchers, with each measurement repeated three times on the dominant foot. Measurements were recorded during both barefoot and with the KW conditions, randomized across participants to mitigate any potential order effects. A 10-s timeout was given to participants between each measurement. The KW was manufactured with a thickness of 3 mm using ethylene vinyl acetate (EVA) with a density of 35 shore A, measuring 8 cm in length and 8 cm in width. [28]. The Kw was placed from second to fifth metatarsal leaving the first metatarsal head free (Fig. 3).

## Statistical analysis

To assess the normality of the distribution of the hallux dorsiflexion resistance data, the Kolmogorov–Smirnov test was used. Considering that the data were not normally distributed (*p* < 0.05), the Wilcoxon test was used to compare hallux dorsiflexion resistance between the barefoot and the KW conditions. Data are expressed as the mean ± standard deviation (± SD).

**Fig. 4:**
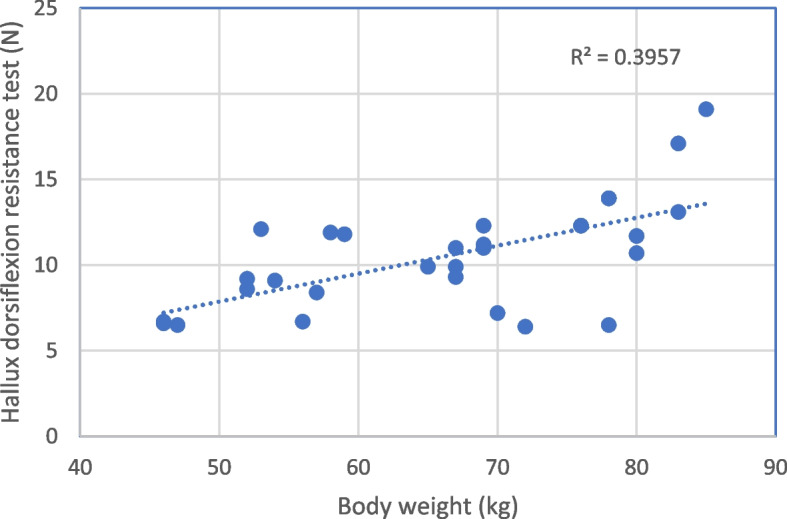
The graph shows Pearson´s correlations between the hallux dorsiflexion resistance test and body mass

Pearson's correlation coefficients and linear regression analyses were employed to assess the association between the hallux dorsiflexion resistance test and body mass (Fig. 4).

## Results

Thirty participants (*n* = 14 women and *n* = 16 men) comprised the sample for this research. The demographic characteristics of the sample are presented in Table 1.

**Table 1:** Demographic data

	**Mean**	**SD**	**Minimum**	**Maximum**
Age (Years)	47.1	14.2	18.0	64.0
Weight (kg)	66.4	12.4	46.0	80.0
Height (Cm)	164.1	6.6	158.0	187.0
FPI (Scores)	2.0	1.5	0	5

*Abreviations*: *SD* standard deviation, *FPI* Foot Posture Index

The hallux dorsiflexion resistance was 19.6 ± 5.2 N during the barefoot condition and it was reduced to 10.5 ± 3.2 N during the KW condition (*p* < 0.001, Cohen’s d effect size = 2.11) (Table 2).

**Table 2:** Hallux dorsiflexion resistance test during barefoot and kinetic wedge conditions

Variables	Barefoot mean ± SD (95% CI)	Kinetic Wedge mean ± SD (95% CI)	p_value Barefoot vs Kinetic Wedge
**Hallux dorsiflexion resistance (N)**	19.6 ± 5.2	10.5 ± 3.2	< 0.001**
	(9.4–30.0)	(6.1–19.1)	

Data are expressed as mean ± SD (95% CI)

*Abbreviations*: *SD* standard deviation, *CI* confidence interval, *p* value level of significance, *p* < 0.05 was considered statistically significant, and *p* < 0.001** was considered strongly statistically significant

Pearson´s correlation coefficient was *r* = 0.63 and the coefficient of determination (r^2^) was 0.396 between the hallux dorsiflexion resistance test and body mass during the barefoot condition.

## Discussion

The present study aimed to determine the effects of the KW on hallux dorsiflexion resistance. The main finding of this research is that the KW reduced the average force required to dorsiflex the 1st MTPJ by 39%. This result suggests that the use of the KW, placed underneath the second to the fifth metatarsal heads, allows a reduction in pressure under the head of the first metatarsal, facilitating the initiation of the windlass mechanism with less force. Becerro de Bengoa et al. [28] foundthat the use of a cut-out beneath the 1st MTPJ induced plantarflexion of the first metatarsal head. The effect of the cut-out orthosis is likely similar to our use of the KW since both scenarios result in a relative lowering of the 1st MTPJ compared to the remaining metatarsophalangeal joints. The passive dorsiflexion of the proximal phalanx of the hallux induces a plantarflexion movement of the first metatarsal head, enabling retrograde force transmission to the first cuneiform and increasing the height of the navicular as well as the supination of the rearfoot [3, 38]. Gatt et al. [9] reported a moderate negative correlation (*r* = -0.534, *p* = 0.004) between the hallux dorsiflexion during the Hubscher maneuver and foot pronation. This correlation implies that as the FPI score increases, the peak dynamic hallux dorsiflexion decreases. Van Gheluwe et al. [39] also indicated that the elevation in pressure under the hallux signifies the activation of a reverse windlass mechanism during walking.

The most prevalent orthopedic devices employed to manage foot pronation are medial wedges positioned under the forefoot and rearfoot. Van Gheluwe and Dananberg [9] demonstrated that these wedges elevate the pressure beneath the first metatarsal head and have the potential to disrupt the initiation of the windlass mechanism. Considering this, KW could perhaps be regarded as the optimal therapeutic choice in cases where there is an increase in pressure beneath the first metatarsal head [39].

Concerning the required force to dorsiflex the hallux during the hallux dorsiflexion resistance test, Moisan et al. [19, 40] quantified the hallux dorsiflexion resistance at 58.3 ± 14.5 N or 6.5% of the mean bodyweight. In comparison, we recorded a lower force of 19.62 ± 5.18 N for the same test. However, the tests were performed using two different tools, namely the Keystone device [19] and a digital force gauge, which could explain the differences across studies. Both methods achieved good to excellent reliability. Further studies should compare both measurements tools for the same population.

To date, we have only identified one prior investigation examining the use of forefoot orthoses devices and their impact on the hallux dorsiflexion resistance test. Sánchez et al. [34] observed that the utilization of a Morton extension below the 1st MTPJ do not increase the required force to dorsiflex the hallux during the hallux dorsiflexion resistance test. It is important to note that our results are not directly comparable, as the orthopedic devices serve distinct functions.

The strengths of this study lie in the enhancement of the windlass mechanism through the use these orthoses devices, resulting in a reduced force required for its initiation. This improvement has the potential to assist individuals in minimizing stress on the plantar fascia or the 1st MTPJ.

Future research directions could involve studying the effects of the KW on the hallux dorsiflexion resistance in clinical populations, such as individuals with plantar fasciopathy or functional hallux limitus. It would also be interesting to study if the hallux dorsiflexion resistance test could help predict the efficacy of orthopedic devices (such as the use of the KW) in patients with foot pathologies and evaluating the evolution of these disorders. Studying how changes in hallux dorsiflexion resistance when using the KW will translate to improvement in the biomechanical gait profile (e.g., kinematic, kinetic and muscle activity) is also needed. Finally, in order to optimize the clinical usefulness of the hallux dorsiflexion resistance test, we urgently need to establish normative values in different ages and sexes.

## Limitations

This study had certain limitations, including issues related to the application of the digital force gauge directly on the skin which could sometime result in a slight slippage. However, the investigators were careful in placing the force applicator on the proximal phalanx of the hallux and all trials with a slight slippage were immediately retaken. It was crucial to consistently verify that the skin was dry and devoid of creams to mitigate this challenge. Another limitation stemmed from the inherent instability observed in certain participants during the test. Finally, we only recruited asymptomatic individuals who rarely use KW in clinical contexts. We suggest being cautious before extrapolating our results to populations with musculoskeletal disorders (e.g., plantar fasciopathy or functional hallux limitus).

## Conclusions

Kinetic wedges reduce the required force to initiate the windlass mechanism during the hallux dorsiflexion resistance test in asymptomatic individuals. Future studies should determine to what extent KW can attenuate the required force to dorsiflex the hallux in individuals with musculoskeletal disorders such as plantar fasciopathy and functional hallux limitus.

## Data Availability

The datasets used and analysed during the current study are available from the corresponding author upon reasonable request.

## Abbreviations

MLA: Medial longitudinal arch
KW: Kinetic wedge
MTPJ: Metatarsophalangeal joint
FPI: Foot posture index
SD: Standard Deviation
CI: Confidence Interval
